# A novel splice-site *FHOD3* founder variant is a common cause of hypertrophic cardiomyopathy in the population of the Balkans–A cohort study

**DOI:** 10.1371/journal.pone.0294969

**Published:** 2023-12-05

**Authors:** Nina Vodnjov, Janez Toplišek, Aleš Maver, Goran Čuturilo, Helena Jaklič, Nataša Teran, Tanja Višnjar, Maruša Škrjanec Pušenjak, Alenka Hodžić, Olivera Miljanović, Borut Peterlin, Karin Writzl

**Affiliations:** 1 Clinical Institute of Genomic Medicine, University Medical Centre Ljubljana, Ljubljana, Slovenia; 2 Biotechnical Faculty, University of Ljubljana, Ljubljana, Slovenia; 3 Department of Cardiology, University Medical Centre Ljubljana, Ljubljana, Slovenia; 4 Faculty of Medicine, University of Ljubljana, Ljubljana, Slovenia; 5 Department of Medical Genetics, University Children’s Hospital, Belgrade, Serbia; 6 Faculty of Medicine, University of Belgrade, Belgrade, Serbia; 7 Clinical Centre of Montenegro, Ljubljanska BB, Podgorica, Montenegro; 8 European Reference Network for Rare, Low Prevalence, or Complex Diseases of the Heart (ERN GUARD-Heart); Shaheed Rajaei Hospital: Rajaie Cardiovascular Medical and Research Center, ISLAMIC REPUBLIC OF IRAN

## Abstract

Founder variants in sarcomere protein genes account for a significant proportion of disease-causing variants in patients with hypertrophic cardiomyopathy (HCM). However, information on founder variants in non-sarcomeric protein genes, such as *FHOD3*, which have only recently been associated with HCM, remains scarce. In this study, we conducted a retrospective analysis of exome sequencing data of 134 probands with HCM for recurrent pathogenic variants. We discovered a novel likely pathogenic variant c.1646+2T>C in *FHOD3* in heterozygous state in eight probands with HCM and confirmed its presence in seven additional relatives. Individuals with this variant had a wide range of ages at onset of the disease (4–63 years). No adverse cardiac events were observed. Haplotype analysis revealed that the individuals with this variant shared a genomic region of approximately 5 Mbp surrounding the variant, confirming the founder effect of the variant. *FHOD3* c.1646+2T>C is estimated to have arisen 58 generations ago (95% CI: 45–81) in a common ancestor living on the Balkans. A founder *FHOD3* c.1646+2T>C variant is the second most common genetic variant in our cohort of patients with HCM, occurring in 16% of probands with a known genetic cause of HCM, which represents a substantially higher proportion than the currently estimated 0.5–2% for causal *FHOD3* variants. Our study broadens the understanding of the genetic causes of HCM and may improve the diagnosis of this condition, particularly in patients from the Balkans.

## Introduction

Hypertrophic cardiomyopathy (HCM) is a common genetic cardiac disease, with an estimated prevalence of one in 200–500 young adults. It is defined by increased left ventricular end-diastolic wall thickness (≥ 15 mm) that cannot be explained by any cardiac, systemic, or metabolic condition capable of causing the observed magnitude of the heart wall thickening. Most HCM are inherited in an autosomal dominant fashion and characterized by variable penetrance and expressivity [1].

Many disease-causing HCM variants are unique for the individual or family and are located in HCM-related genes [2]. Still, in a specific population, founder variants may account for a substantial portion of disease-causing variants among probands [3, 4]. So far, HCM-causing founder variants have been reported in *MYBPC3* [4], *MYH7* [5], *TPM1* [6, 7], *JHP2* [8], and *TNNI3* [9].

Most of the HCM-causing variants are located in genes encoding sarcomeric proteins [1]. However, other genes involved in the development and maintenance of sarcomeric filaments also play an important role [2]. One of the genes recently associated with HCM is *FHOD3* [10–13].

FHOD3 belongs to the formin protein family and plays an essential role in the sarcomere organisation of the cardiomyocytes through its actin assembly activity. It contains multiple domains, including formin homology domains (FH1, FH2, FH3), GTPase binding domain (GBD), diaphanous inhibitory domain (DID) and diaphanous auto-regulatory domain (DAD). The intermolecular interaction between DID and DAD leads to dimerization of FHOD3, which promotes the synthesis of actin filaments [14].

*FHOD3* is expressed in various human tissues and undergoes alternative splicing. The longer isoform, which contains the exon 12, is primarily abundant in the heart. Cardiac-specific exon 12 of the *FHOD3* encodes a region that is required for the localisation of the long FHOD3 isoform to sarcomeric C-zone, a process mediated through interaction between FHOD3 and cardiac myosin-binding protein C. The precise location of the long FHOD3 protein isoform is essential for the proper cardiac function [15].

Disease-causing variants in *FHOD3* are most commonly associated with the phenotype of HCM, with penetrance being incomplete [11, 16, 17], but the clinical picture can develop even in childhood [16, 17]. In some patients, the criteria for a diagnosis of left ventricular non-compaction cardiomyopathy have been fulfilled [11, 18], while the role of rare *FHOD3* variants in dilated cardiomyopathy remains as yet unproven [19]. Most pathogenic *FHOD3* variants associated with HCM so far seem to be restricted to non-truncating variants (missense, splice-site, and in-frame deletions/insertions variants) and cluster to two DID regions, to exon 12 and to the coiled-coil domain encoded by exons 15 and 16 [11, 18, 20–23].

The aim of the present article was to clinically characterise the cardiac phenotype associated with *FHOD3* c.1646+2T>C in individuals of the Balkan origin. We also determined the surrounding haplotype of the variant, and estimated it’s time of origin.

## Results

### Recurrent *FHOD3* variant in the study population—genotype analysis and variant classification

We retrospectively screened our exome sequencing database for recurrent variants in 134 probands with a suspected hereditary form of HCM, mostly inhabitants of Slovenia, and identified the genetic cause in 51 patients (38%). Eight (16%) probands with genetically confirmed HCM were identified with a splice site variant in *FHOD3*(NM_001281740.3): c.1646+2T>C. Through cascade screening of four accessible families, we identified additional seven relatives with the *FHOD3* variant, five of whom had clinical evidence of HCM.

The *FHOD3* c.1646+2T>C variant is located at the second donor splice-site after exon 12. *FHOD3* c.1646+2T>C is absent from the gnomAD population database [24], an internal Slovenian Database (more than 10,000 exomes/genomes), the TopMed database [25, 26] and has been reported twice as a variant of uncertain significance in the ClinVar database (ID: 1862304). Online tools for predicting the effect of the variant on splicing indicated its damaging effect (SpliceAI 0.90 for donor loss, ADA score 0.807755, VarSeak class 5). Various splice-site variants (c.1646+1G>A/C/T), localized at the first donor splice site after exon 12, have been recurrently identified in patients with HCM [11, 18, 23]. These variants are predicted to cause skipping of the symmetrical exon 12, leading to a 120 amino acids long in-frame deletion from p.Ser429 to p.Ser549 [11, 23] and appear to act through a dominant negative effect [18]. Notably, for none of the reported variants, functional characterization has been performed to date.

Following ACMG/AMP standards and guidelines for interpretation of sequence variants [27] modified by ACGS recommendations [28], the variant was classified as likely pathogenic (criteria applied: PS4_STR, PP1_SUP, PP3, PM2).

### The phenotype of the individuals with a recurrent *FHOD3* variant

A heterozygous *FHOD3* c.1646+2T>C variant was observed in fifteen individuals; eight probands and seven relatives. Eleven of them had clinical evidence of HCM, two showed preclinical characteristics of HCM, and two were asymptomatic for the disease (Table 1, S1 Table).

**Table 1:** Clinical characteristics of 13 individuals with heterozygous *FHOD3* c.1646+2T>C who showed clinical evidence of HCM at first evaluation.

Characteristics (n = 13)	Mean±st.dev (min-max)	No. of individuals meeting criteria/all individuals
**Gender [% of men]**	46	
**Age at diagnosis [years]**	32±18 (4–63)	
**Family history for SCD, aSCD**		2/8
**NYHA Classification**		
Class I		6/12
Class I-II		4/12
Class II		1/12
Class III		1/12
**Symptoms at diagnosis**		
dyspnoea after exertion		2/12
chest pain		4/12
fatigue		2/12
palpitations		3/12
syncope		2/12
none		6/12
**MLVWT [mm]**	21±7 (11–31)	
**Presence of outflow obstruction**		0/12
**LAVI [mm]**	39±9 (25–52)	
**LAD [mm]**	38±8 (28–50)	
**LV filling pattern**		
I (mildly impaired)		5/8
II (moderately impaired)		3/8
**EF at first evaluation [%]**	67±9 (58–80)	
**Presence of LV apical aneurysm**		0/12
**sPAP [mm Hg]**	25±4 (22–31)	
**Presence of AF**		0/13
**Presence of NSVT**		3/13
**Presence of ICD**		2/13

SCD, sudden cardiac death; aSCD, aborted sudden cardiac death; NYHA, New York Heart Association; Class I, “no symptoms”; Class II, “mild symptoms”; Class III, “marked limitations due to symptoms”; MLVWT, maximal left ventricle wall thickness; LAVI, left atrial volume index; LAD, left atrial diameter; LV, left ventricle; EF, ejection fraction; sPAP, systolic pulmonary artery pressure; AF, atrial fibrillation; NSVT, non-sustained ventricular tachycardia; ICD, implantable cardioverter-defibrillator.

The individuals’ age at diagnosis of the disease ranged from 4 to 63 years of age (mean age 32±18). There was an equal representation of men and women (46% of men). The mean age of the males (13 years) was significantly lower (p = 0.009) than the mean age of the females (42 years). Half of the individuals were asymptomatic at diagnosis, while others reported chest pain, dyspnoea on exertion, fatigue, palpitations and syncope. At initial evaluation, the individuals presented mild to severe left ventricular thickening of the left ventricular wall (11–31 mm). Enlargement of the left atrium was observed in three individuals and the presence of fibrosis was observed in two individuals. Left outflow obstruction, severely impaired left ventricular filling pattern, apical aneurysm, reduced left ventricular ejection fraction, and atrial fibrillation were not observed. No adverse cardiac events, such as sudden cardiac death and aborted sudden cardiac death, were observed. One individual had an additional genetic diagnosis of Alström syndrome.

In addition, one first-degree relative suffered an aborted sudden cardiac death at the age of 51 and another died suddenly at the age of 64. None of them have undergone genetic testing. Two relatives, a 22-year-old male and a 41-year-old female with the *FHOD3* c.1646+2T>C variant, had no clinical signs of the disease observed on TTE and had no HCM-related symptoms. Additional information is available in the Supporting information.

### Haplotype investigation

To investigate whether individuals share a common ancestor, the surrounding sequence of the variant was analysed using five microsatellite markers, two dinucleotide repeats and 16 reference SNP markers in 11/15 individuals for whom sufficient DNA concentration was available. A shared haplotype, rs1350390(C)-D18S456(17)-rs4799705(G)-rs4270249(C)-D18S1135(18)-rs1383290(A)-rs355318()-rs579596(T)-variant(C)-rs483351(T)-rs480345(G)-D18S1102(17)-D18S475(20)-rs1196588(A), spanning approximately 5 Mb around the variants was observed (Table 2). The finding supports the hypothesis that c.1646+2T>C has a founder role among individuals. A careful screening of the family history of the individuals revealed that all had ancestors from Bosnia, Serbia and Monte Negro.

**Table 2:** Haplotype marker analysis in 11 individuals with *FHOD3* c.1646+2T>C variant.

Marker ID	REF	ALT	REP	P1	P1.1	P2	P3	P3.1	P4	P4.1	P4.2	P5	P6	P7	HAP
**rs11660224**	A	C		A/C	A/C	C/C	A/A	A/C	A/C	A/C	A/A	A/A	A/A	A/A	/
**D18S47**			AC	17/19	17/19	19/20	17/17	17/17	17/19	17/19	17/18	17/17	17/17	17/19	/
**rs628064**	A	T		A/T	A/T	T/T	A/A	A/A	A/T	A/T	A/T	A/A	A/A	A/T	/
**rs1350390**	T	C		T/C	T/C	C/C	T/C	C/C	C/C	C/C	C/C	C/C	C/C	T/C	C
**D18S456**			TG	17/18	17/-	17/17	17/18	17/16	17/16	17/16	17/17	17/16	17/16	17/18	17
**rs4799705**	G	T		G/G	G/G	G/T	G/G	G/G	G/G	G/G	G/T	G/G	G/G	G/G	G
**rs4270249**	A	C		C/C	C/C	C/A	C/A	C/A	C/C	C/C	C/C	C/C	C/A	C/C	C
**D18S1135**			AC	18/23	18/23	18/18	18/20	18/18	18/18	18/18	18/19	18/19	18/19	18/19	18
**rs1383290**	A	C		A/C	A/C	A/C	A/C	A/C	A/C	A/C	A/C	A/C	A/A	A/A	A
**rs355318**	T	A		T/T	T/T	T/A	T/T	T/A	T/A	T/A	T/A	T/A	T/T	T/T	T
**rs579596**	T	C		T/C	T/C	T/C	T/C	T/T	T/C	T/C	T/C	T/T	T/T	T/T	T
**variant**	T	C		T/C	T/C	T/C	T/C	T/C	T/C	T/C	T/C	T/C	T/C	T/C	C
**rs483351**	C	T		C/T	C/T	C/T	C/T	T/T	C/T	C/T	T/T	C/T	C/T	C/T	T
**rs480345**	G	A		G/A	G/A	G/A	G/G	G/G	G/A	G/A	G/G	G/G	G/G	G/A	G
**D18S1102**			TG	23/17	17/-	23/17	17/21	17/25	23/17	23/17	17/25	23/17	23/17	17/24	17
**D18S475**			TG	20/20	20/20	20/21	20/23	20/18	20/18	20/18	20/20	20/19	20/20	20/20	20
**rs1196588**	G	A		G/A	G/A	A/A	G/A	A/A	G/A	G/A	G/A	A/A	A/A	G/A	A
**VNTR1**			AC	18/14	14/-	14/20	14/21	18/14	14/16	14/16	14/14	22/22	14/21	14/16	/
**VNTR2**			GT	18/15	15/18	20/20	18/15	18/18	18/16	18/16	16/18	15/15	18/15	15/15	/
**rs976314**	T	C		T/C	T/C	T/C	T/C	T/T	T/C	T/C	T/C	C/C	T/C	C/C	/
**rs925238**	A	G		A/G	A/G	G/G	A/G	A/A	A/G	A/G	A/A	A/G	A/G	A/G	/
**rs376716**	C	T		C/T	C/T	T/T	C/T	C/T	T/T	T/T	T/T	C/T	C/T	T/T	T
**rs1367689**	A	G		G/G	G/G	A/A	A/A	A/A	G/G	G/G	G/G	G/G	G/G	G/A	/
**rs1433936**	A	T		A/T	A/T	A/T	A/T	A/T	A/A	A/A	A/T	A/A	A/T	A/T	/

REF, reference allele; ALT, alternative allele; REP, repeat; PX (X = numeric identifier of a family), individual; PX.X, relative of a family X; HAP, haplotype. SNP markers are given as allele1/allele2 observed at the locus. Repeat markers are denoted as the number of repeats per allele1/allele2 observed at the locus.

### Age estimation of *FHOD3* c.1646+2T>C

**Fig 1:**
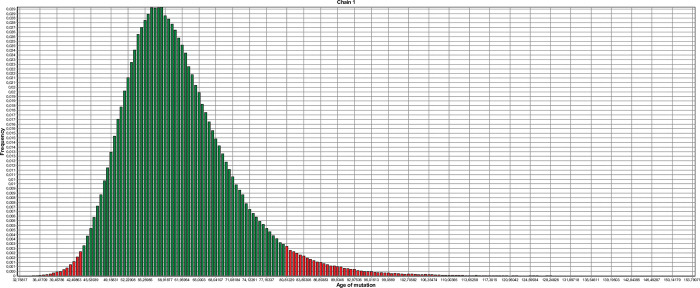
The estimated age of *FHOD3* c.1646+2T>C variant.

Haplotype analysis suggested that a *FHOD3* c.1646+2T>C in individuals originates from the common ancestor. Using Bayesian methods on genetic data from 11 individuals, we estimated that the variant was introduced into the Balkan population around 58 generations ago, with a 95% confidence interval between 45 and 81 generations (Fig 1). Assuming each generation to last 20 years, this indicates that the variant arose around 1160 years ago (95% CI: 900–1620 years).

(X-axis) The age of the variant is expressed in generations. (Y-axis) Frequency of each estimation out of a total of 1,000,000 iterations. Green bars, 95% confidence interval. The highest frequency was detected for about 58 generations while 95% CI spans from 45 to 81 generations.

## Discussion

Pathogenic variants in *FHOD3* are estimated to be causal for HMC in 0.5–2% of patients with known genetic aetiology of HCM [2]. In our cohort, we have detected a novel recurrent heterozygous likely pathogenic *FHOD3* c.1646+2T>C variant in 16% of probands with genetically confirmed form of HCM. This variant is the second most common genetic cause of HCM among patients with HCM who have been genetically tested at our institute, after the founder variant c.913_914del in *MYBPC3*. The proportion of patients with HCM, carrying the disease-causing variant in *FHOD3*, is much higher in our cohort compared to other studies [2, 11]. *FHOD3* was only recently added to the list of intrinsic genes implicated in HCM in 2022 [2]. Therefore, it has not been included in earlier studies using panel sequencing to elucidate the genetic background of patients with HCM [29–31]. With the inclusion of the *FHOD3* in cardiomyopathy gene panels, the prevalence of *FHOD3*-associated HCM could be significantly higher than currently estimated, particularly among patients with HCM with Balkan ancestry.

### Phenotype characteristics of the individuals with founder *FHOD3* variant

In our study, we found that the onset of the disease varied greatly between individuals with the *FHOD3* founder variant. Five individuals (38%) (including one with Alström syndrome) had clinical manifestations of HCM in the second decade of life, and the youngest individual in our cohort was diagnosed at four years of age. Although the first large cohort reporting patients with HCM and an identified *FHOD3* causal variant consisted mainly of adult probands [11], other studies have suggested that a severe HCM phenotype may be present already in children [16, 17].

We observed that half of the individuals in our cohort had no clinical symptoms at the time of diagnosis, and none experienced an adverse cardiac event or reached the disease endpoint. This may be due to the young age of the individuals in the study cohort and the short duration of follow-up. In the largest study published to date, the phenotype of patients with *FHOD3* variants was reported to be as relatively mild, with a low rate of adverse events in young patients and a cardiovascular endpoint rate similar to that in unselected HCM cohorts [11]. These findings were not replicated by Wu et al. (2021), who reported a more severe disease course in Chinese patients with *FHOD3* variants, with a significant proportion experiencing sudden cardiac death or reaching the disease endpoint [22]. Incomplete penetrance of the disease in variant carriers was observed in both studies [11, 22] and replicated in our cohort. In addition, LV hyper-trabeculation was noted in two individuals, which is consistent with observations [11, 18].

We also observed a variable expression of clinical manifestations between patients, which is a common feature of HCM. Interestingly, the phenotype of HCM was not identical even in monozygotic twins with the *FHOD3* founder variant found in our study. The clinical picture of HCM was present in both, but the time of onset of symptoms, septal thickening and left ventricular ejection fraction were different (S1 Table).

Furthermore, a marked gender difference in the age of diagnosis was observed by Ochoa et al. (2018) [11]. They showed that, on average, males were diagnosed 10 years earlier than females, but this finding was not replicated by Wu et al. (2021) [22]. Gender-dependent differences were also observed in our cohort, where the mean age of male with the variant was significantly lower than the mean age of female with the variant (20 years and 42 years, respectively) (p = 0.02). This remarkable difference could be due to the small sample size and the contribution of other genetic factors, for example the individual with a double diagnosis (Alström syndrome and HCM) was male. Finally, both previous studies reported that more male individuals harbouring the *FHOD3* candidate variant have the HCM phenotype [11, 22], which was not observed in our study.

### Haplotype size and age estimations

Founder variants have previously been reported in patients with HCM in diverse populations [3, 4], being identified in up to 58% of patients with HCM in certain populations [3]. No founder variant has yet been reported in *FHOD3*.

Genetic analysis of 11 individuals with *FHOD3* c.1646+2T>C revealed that the variant is positioned on a single haplotype of approximately 5 Mb in length. The estimated haplotype spans the region between 30.9 Mb and 35.9 Mb on chromosome 18 (hg19 assembly). Examination of the three-generation family history of the individuals suggested that the common ancestor originates from the Balkans. A careful screening of the family history of the individuals revealed that all had ancestors from Bosnia, Serbia and Monte Negro. Therefore, we hypothesize that a common ancestor had lived in the population of the Balkans.

The age of origin of *FHOD3* c.1646+2T>C was estimated to be approximately 1160 years ago (95% CI: 900–1640 years). The age estimation, given by DMLE+ software, is relatively broad since confidence intervals cover the era of more than 700 years. Accuracies of chosen parameters, such as growth rate and marker selection [32], as well as factors such as demographic changes, selection, recombination, and disappearances/reoccurrence of the same mutation in unrelated individuals [33] substantially impact the estimated age. Since we hypothesized that the variant was introduced into the Slovenian population, the estimated age of origin of the variant might be different than estimated.

## Conclusion

To sum up, we report a novel founder heterozygous variant in *FHOD3* (c.1646+2T>C), causal for a hereditary form of hypertrophic cardiomyopathy. Fifteen individuals with a variant from eight families were identified. The variant shows incomplete penetrance, highly variable disease onset in affected individuals, and can cause HCM-related cardiac changes already in childhood. The analysis revealed a haplotype of approximately 5 Mb shared by the individuals having the variant. Examination of the family history revealed that the variant most likely arose in a common ancestor, who had lived on the Balkans. Additional studies would be needed to better understand the exact mechanism of the variant’s pathogenicity, its contribution to the phenotype, the variant’s penetrance and long-term outcomes.

### Study limitations

The splicing effect of the c.1646+2T>C variant in *FHOD3* could not be determined in this study. PCR-based RNA diagnostics [34] were performed on clinically available control samples (blood and fibroblasts), which showed the presence of the PCR product of *FHOD3* exclusively in fibroblasts. Unfortunately, due to the retrospective nature of the study, patient fibroblasts were not available. Furthermore, the establishment of in vitro cell or animal models to validate the pathogenicity of the variant was hampered by financial constraints.

## Materials and methods

We used exome sequencing data collected during routine diagnostic testing of patients referred to our institute for suspected hypertrophic cardiomyopathy. We searched for common variants within the patient cohort and identified the *FHOD3* c.1646+2T>C. To investigate the origin of the variant, we performed haplotype analysis and calculate the estimated age. Additionally, we collected and analysed the clinical characteristics of individuals carrying the variant.

### Study population

The study included 134 probands with HCM, who were referred to the Clinical Institute of Genomic Medicine (CIGM), Ljubljana, Slovenia, between the years 2010 and 2022 to be screened for the genetic cause of the disease as part of routine diagnostic procedures. Probands were mainly Slovenian inhabitants and a smaller fraction of them were from the Balkans countries. HCM was diagnosed according to the AHA/ACC Guideline for the Diagnosis and Treatment of Patients with Hypertrophic Cardiomyopathy [1]. Written informed consent was obtained from all the participants. The study was approved by a National Medical Ethics Committee, Republic of Slovenia (0120-71/2022/3) and was performed in concordance with the Declaration of Helsinki. As part of routine diagnostic procedures, individuals were anonymised with identification codes. The data were accessed for research purposes between 01/02/2023 and 01/05/2023.

### Sequencing and bioinformatics analysis

All individuals underwent next-generation sequencing (NGS) genetic testing, which was performed as follows. Between January 2010 and 2013, 14 individuals underwent cardiomyopathy panel sequencing at an external laboratory (GENDIA—Genetic Diagnostic Network, Antwerp, Belgium). Between January 2014 and July 2019, 27 individuals underwent clinical exome sequencing and between July 2019 and December 2022, 93 individuals underwent exome sequencing. Sequencing and data analysis were performed at our institute as previously described [35–37]. For clinical and exome sequencing, the median minimum exome coverage was 60x, with more than 95% of the targets covered with at least 10x sequencing depth.

Individuals who underwent panel and clinical exome sequencing were not screened for variants in *FHOD3* during the initial analysis, as *FHOD3* was not added to the list of genes intrinsically linked to HCM until 2022 [2]. After *FHOD3* c.1646+2T>C was observed in five individuals in the cohort who had undergone exome sequencing, additional screening for the variant was carried out in individuals who had previously undergone panel or clinical exome sequencing using the Sanger sequencing method. Briefly, the region containing the variant was amplified using a set of primers (forward primer: 5’ CTGAAGGTGTCACCGAC-CAT 3’, reverse primer: 5’ CACCTGGCTTGGTACAAGATGT 3’). The size of the PCR product was 452 bp. The sequencing data were analysed using Geneious® software version 10.2.6. In addition, the variant was identified in three individuals who had previously undergone panel or clinical exome sequencing and in a total of seven relatives. Fifteen individuals found to have *FHOD3* c.1646+2T>C were included in the next steps of this research.

To examine if individuals with a heterozygous *FHOD3* c.1646+2T>C share a common ancestor, whole genome sequencing (WGS) was performed for 11 individuals with the variant. For four individuals, WGS was not possible due to the very low concentration of DNA in the sample. Sequencing and data analysis were done as previously described by Bergant et al. (2021) [38]. For a relative from Family 1, PCR-based amplification of DNA was done before WGS because of the low DNA concentration in the sample.

### Clinical evaluation and family screening

During the pre-genetic testing counselling, we collected the cardiac data obtained during the individuals’ initial evaluation. When available, data on resting 12-lead ECG, transthoracic echocardiography (TTE), cardiac magnetic resonance imaging (MRI), 24/48-hour Holter monitoring, exercise testing, and biochemical laboratory tests were gathered. The three-generation family tree was constructed to study the segregation of the variant with the phenotype. Relatives were invited for genetic testing and asked for reports of cardiac examination.

Summary statistics 13 individuals with *FHOD3* c.1646+2T>C exhibiting HCM characteristics are presented as number of individuals for categorical variables and mean ± standard deviation and range in brackets for continuous variables. The Kolmogorov-Smirnov normality test was used to test the normality of the data distribution. Additional descriptions of the cardiac characteristics of individuals are provided in the Supporting Information.

### Haplotype estimation

Annotated genomic data were uploaded to the program IGV [39]. Markers, located in *FHOD3* and its surrounding, were manually screened for a common haplotype among all the individuals with the variant. Linkage analysis was done using five microsatellite markers (D18S1102, D18S1135, D18S456, D18S47, D18S475), two di-nucleotide repeats, and 16 reference SNPs (rs11660224, rs628064, rs1350390, rs4799705, rs4270249, rs1383290, rs355318, rs579596, rs483351, rs480345, rs1196588, rs976314, rs925238, rs376716, rs1367689, rs1433936). All SNPs used in the analysis had a minor allele frequency (MAF) in the European non-Finnish population in the range 0.43–0.60. Di-nucleotide repeats (VNTR1, VNTR2) are located at 36278972–36279007 and 36388746–36388785 sites in reference hg19 genome, having 18 AC and 20 GT repeats on reference genome build, respectively. Markers cover an approximately 10 Mb wide region. The most probable haplotype was reconstructed. A phase of the haplotype was reconstructed based on segregation analysis of variants between family members.

### Age estimation of *FHOD3* c.1646+2T>C

The DMLE+2.3 software was used to estimate the age of *FHOD3* c.1646+2T>C origin in probands with the estimated haplotype. The program utilizes the Markov Chain Monte Carlo algorithm (MCMC) for Bayesian appraisal of variant age based on the linkage disequilibrium observed in multiple genetic markers [40]. Here, SNPs and VNTRs (rs3786309, rs1350390, VNTR1, VNTR2), flanking the boundaries of the estimated haplotype, were used. One million iterations of the MCMC were employed to construct the posterior distribution histogram results. The population growth rate was set as 0.12 based on the formula described before [32, 41]. Present population (Pp) size was defined as the number of inhabitants of Slovenia (n = 2110547) [42], past population size (Po) as an estimated number of inhabitants in 1857 (n = 1101854) [43] and a generation interval of 20 years was considered. The disease sample ratio of 0.0011 was determined; considering all individuals for which haplotype analysis was done (n = 11) and all individuals (n = 7901) reported in an internal Slovenian Database on 2^nd^ of March 2023. To determine map distances between markers in Morgans, an assumption of 1 cM ~ 1 Mbp was used [44]. Haplotypes of the healthy population were generated by splitting the genotyped data of all the individuals into every possible haplotype with a frequency of 1.

## Supporting information

S1 File Clinical characteristics of individuals with c.1646+2T>C in *FHOD3*. (DOCX)

S1 Table Summary of clinical characteristics of individuals with c.1646+2T>C in *FHOD3*. SCD, sudden cardiac death; NYHA, New York Heart Association; Class I, “no symptoms”; Class II, “mild symptoms”; Class III, “marked limitations due to symptoms”; MLVWT, maximal left ventricle wall thickness; LAVI, left atrial volume index; LAD, left atrial diameter; LV, left ventricle; EF, ejection fraction; sPAP, systolic pulmonary artery pressure; AF, atrial fibrillation; NSVT, non-sustained ventricular tachycardia; ICD, implantable cardioverter-defibrillator; N/A, not available.
(XLSX)
