# Supplementary material for: A novel splice-site FHOD3 founder variant is a common cause of hypertrophic cardiomyopathy in the population of the Balkans–A cohort study
Source: PLoS One. 2023 Dec 5;18(12):e0294969. doi: 10.1371/journal.pone.0294969 (PMC10697513; doi:10.1371/journal.pone.0294969)
Supplement: S1 File — (DOCX) [file pone.0294969.s001.docx]

**Supplementary material**

*Clinical characteristics of the probands and their relatives*

***Family 1.*** A proband from family 1 (P1) was examined at the age of 35 years because of a positive family history of heart disease. The ECG was abnormal and a TTE was performed, which showed a severely thickened interventricular septum (IVS) of up to 27 mm and mild diastolic dysfunction. Cardiac MRI showed that at least 10 mm of the IVS thickening was due to trabeculation of the right ventricle. Late gadolinium enhancement (LGE) signal was present in the inferior segment of the left ventricle (LV) and the right ventricle (RV). Exercise testing showed mildly reduced exercise capacity (79%). NT-proBNP was elevated (3566). Holter monitoring at the time of diagnosis of HCM showed no significant arrhythmias, whereas bifascicular block was reported four years later. She reported no other cardiac problems and is not on any cardiac-related therapy. The proband's mother, who lives abroad, reportedly suffered two myocardial infarctions after the age of 40, was diagnosed with HCM and had an ICD implanted. The proband's sister (P1.1), who also lives abroad, was diagnosed with HCM at the age of 44 and had an ICD implanted due to arrhythmias. Genetic screening of the sister revealed the presence of a heterozygous *FHOD3* c.1646+2T>C.

***Family 2.*** A proband from family 2 (P2) underwent TTE for suspected myocarditis at the age of 31 years. The TTE showed mildly increased wall thickness up to 11 mm, but no other structural abnormalities of the heart. Increased wall thickness, elevated NT-proBNP level (496) and relatively young age (31 years) were the reasons for referral for genetic testing, which revealed heterozygous *FHOD3* c.1646+2T>C. The patient is asymptomatic and not receiving any treatment. Segregation analysis of the variant in other first-degree family members was declined.

***Family 3.*** In a proband from family 3 (P3), the first signs of heart disease were detected at the age of 23 years, when an unusual ECG recording was obtained, but no further investigations were carried out at that time. At the age of 39, a TTE revealed a severely thickened IVS of up to 37 mm. She reported palpitations but attributed them to stress. At age 42, TTE showed an asymmetric thickened IVS up to 32 mm, normal LV size (LVEDVI 61 ml/m) and ejection fraction (66%), severely reduced LV global longitudinal strain (-10.0%), mild to moderate diastolic dysfunction, no obstruction, elevated LV end-diastolic pressure, moderately enlarged left atrium (LAD = 41 mm), normal right atrium and normal valves. Cardiac MRI confirmed the findings and also showed structural myocardial changes with contrast enhancement in the inferoseptal segment of the heart, which was thought to be the substrate for the arrhythmia. Normal physical activity without cardiac restriction (108%) was observed during the exercise test. Sixty-six hours of Holter monitoring showed mildly increased ectopic activity in the form of NSVTs lasting up to 6 beats and polymorphic ventricular extrasystoles (VES). There was an elevated blood level of NTproBNP (2409). Based on these findings, an ICD was implanted for primary prevention and cardiac medical therapy was prescribed.

The proband's father (P3.1) had an abnormal ECG recording at the health screening. He reported no cardiac symptoms. TTE at the age of 54 years showed normal LV size, asymmetric thickening of the IVS up to 19 mm, mild to moderate diastolic dysfunction and mild enlargement of the left atrium (LAD = 50 mm). Holter monitoring showed increased ectopic activity and supraventricular extrasystoles (SVES) occurring in pairs, triplets and prolonged episodes (up to 22 beats). He had been treated for hypertension and hyperlipidaemia for 20 years. Genetic testing revealed the presence of a heterozygous *FHOD3* c.1646+2T>C.

The proband has two sons who were 16 and 22 years old at the time of genetic screening. Genetic analysis revealed the presence of heterozygous *FHOD3* c.1646+2T>C in both. The older son is asymptomatic and the TTE did not show any characteristic features of HCM. The younger son was a recreational athlete and reported occasional palpitations during exercise. At the age of 15 years, TTE showed mild thickening of the LV wall up to 12 mm, but no other cardiac abnormalities were observed.

***Family 4.*** The proband of family 4 (P4) was referred for genetic testing after an abnormal ECG was observed during a medical examination and a positive family history at the age of 63 years. The proband reported some palpitations and mild exertional dyspnoea at the time of diagnosis of HCM. TTE showed a normal-sized left ventricle, hypertrophied anterior segment of the IVS up to 18 mm, severely enlarged left atrium and moderate diastolic dysfunction. Cardiac MRI showed thicker basal anterior and basal anteroseptal segments of the LV, smaller ventricles, enlarged left atrium and preserved systolic function (LVEF 58%). Higher levels of contrast enhancement were present in the basal segment of the heart, indicating the presence of fibrosis. Exercise stress testing showed normal performance and no pathological cardiac abnormalities. Holter monitoring showed 200 SVES and cardiac therapy was prescribed. The subject's son died during an activity. Post-mortem examination revealed HCM with IVS thickened to 18 mm and enlarged left atrium (LAVI = 45-50 mL/m2). Gene analysis was not possible.

The proband also has a monozygotic twin with HCM (P4.1). The twin sister was diagnosed with HCM at the age of 58 following a cardiac evaluation for dyspnoea and chest pain. An ECG showed a prolonged QT interval. TTE showed a hypertrophied IVS of 21 mm, mildly reduced LV volumes and an impaired left ventricular filling pattern. No left ventricular outflow obstruction, SAM or valvular or aortic abnormalities were reported. She is on cardiac therapy. She has one daughter (P4.2) with a detected heterozygous *FHOD3* c.1646+2T>C who was phenotypically asymptomatic at the age of 41 years.

***Family 5.*** A proband from family 5 (P5) was referred for genetic testing because of pigmentary retinopathy, hearing loss, obesity and HCM at the age of 15 years. TTE showed asymmetric HCM with enlarged LV and LV hyper-trabeculation. Cardiac MRI showed HCM with mild diastolic dysfunction and patches of myocardial fibrosis after contrast administration. Genetic testing revealed the presence of a homozygous pathogenic variant in ALMS1, consistent with a diagnosis of Alström syndrome, and a second variant, the heterozygous *FHOD3* c.1646+2T>C, which is thought to contribute to an early hypertrophic cardiomyopathy phenotype.

***Family 6.*** A proband from family 6 (P6) was referred for genetic testing after being diagnosed with HCM and suspected of having skeletal myopathy at the age of 15, and because of a positive family history of SCD. TTE showed thickening of the IVS to 29 mm and the posterior wall of the LV to 17 mm. ICD implantation was suggested, but the patient refused. His father had died suddenly of myocardial infarction at the age of 64. Genetic analysis of the father was not possible. Exome sequencing did not identify a genetic cause of skeletal myopathy in the proband.

***Family 7.*** In the proband of family 7 (P7), HCM was discovered incidentally during a TTE performed after the proband had received a blow to the chest at the age of 16. The TTE showed normal-sized atria, a thickened IVS of 16 mm, an LV end-diastolic diameter of 54 mm and a bicuspid aortic valve. It was reported that the subject's grandmother had died of a heart defect (age unknown), but genetic analysis of her or the subject's parents was not possible.

***Family 8.*** A proband from family 8 was referred for genetic testing after being diagnosed with hypertrophic cardiomyopathy at the age of 4 years and having a positive family history for HCM. The proband was asymptomatic at the time of HCM diagnosis. TTE showed a normal cardiac structure with the exception of a thickened left ventricular endocardium around the papillary muscles and partially periapically. Holter monitoring showed no arrhythmias.

The proband's mother was diagnosed with cardiomyopathy at the age of 28 years after experiencing dizziness, chest pain, palpitations and more syncopal events. TTE showed no other structural cardiac abnormalities, with an IVS thickness of 23 mm and a posterior wall thickness of 16 mm. Holter monitoring showed multiple VES and an episode of tachycardia with wide QRS complexes. Based on these findings, an ICD was implanted. Genetic testing of the mother revealed the presence of a heterozygous *FHOD3* c.1646+2T>C.
